# Engineering Polymeric Biomaterials for Radiation-Induced Vaginal Injury After Cervical Cancer Therapy: Pathobiological Basis, Material Strategies, and Future Perspectives

**DOI:** 10.3390/bioengineering13101169

**Published:** 2026-10-08

**Authors:** Hui Chang, Xiaolong Wang, Yutong Wu, Qian Li, Qingsen Gao, Xianhu Liu, Chenchen Ren

**Affiliations:** 1 Tianjian Laboratory of Advanced Biomedical Sciences, Department of Gynecology, The Third Affiliated Hospital of Zhengzhou University, Zhengzhou University, Zhengzhou 450052, China; 2 National Clinical Research Center for Obstetrics and Gynecology, Henan Branch, Zhengzhou 450052, China; 3 Zhengzhou Key Laboratory of Cervical Diseases, Zhengzhou 450052, China; 4 Institute of Polymer Materials, Friedrich-Alexander University Erlangen-Nuremberg, 91058 Erlangen, Germany; 5 National Engineering Research Center for Advanced Polymer Processing Technology, Zhengzhou University, Zhengzhou 450002, China

**Keywords:** vaginal stenosis, pelvic radiotherapy, mucoadhesive hydrogels, vaginal dilators, decellularized extracellular matrix, tissue engineering

## Abstract

Radiation-induced vaginal injury is an underrecognized complication of cervical cancer radiotherapy and evolves through a pathological continuum involving epithelial depletion, oxidative stress, chronic inflammation, microvascular dysfunction, extracellular-matrix remodeling, fibrosis, loss of tissue compliance, and vaginal stenosis. Current management remains largely supportive and is constrained by short local residence, limited anatomical adaptability, variable adherence, and insufficient control of radiation-specific tissue damage. This Mini-Review examines polymeric biomaterials for the prevention and repair of radiation-induced vaginal injury from a pathobiology–material–function perspective. Three complementary strategies are discussed: local polymeric formulations and mucoadhesive hydrogels for mucosal protection and sustained delivery; personalized dilators, stents, and adaptive devices for maintaining vaginal patency; and regenerative scaffolds and tissue-engineered constructs for restoring epithelial, vascular, stromal, and smooth-muscle compartments. Conventional hyaluronic-acid formulations have progressed furthest clinically, whereas responsive hydrogels, shape-adaptive devices, extracellular-matrix-derived materials, and cell- or exosome-based scaffolds remain predominantly preclinical. Importantly, much of the evidence derives from acute irradiation models, non-irradiated reconstruction studies, or engineering prototypes and therefore does not yet demonstrate durable reversal of chronic fibrosis or stenosis. Future development should prioritize stage-specific multifunctional interventions, fractionated irradiation models, standardized structural and functional outcomes, and rigorous oncological safety assessment. Acellular hydrogels, cell-free hybrid scaffolds, and removable drug-eluting devices may offer the most practical near-term translational routes.

## 1. Introduction

Cervical cancer remains a major threat to women’s health despite being largely preventable through human papillomavirus vaccination, effective screening, and timely treatment of precancerous lesions [1]. Approximately 604,000 new cases and 280,000 deaths were estimated worldwide in 2024, with the greatest burden occurring in regions where access to prevention, early diagnosis, and high-quality treatment remains insufficient [2]. As a result, cervical cancer continues to affect a substantial population of women requiring multidisciplinary oncological treatment and long-term follow-up. Although improvements in prevention and tumor control remain fundamental, increasing survival has also highlighted the need to address treatment-related morbidity, functional recovery, and quality of life among cervical cancer survivors.

The management of cervical cancer includes surgery, radiotherapy, systemic therapy, or combinations of these approaches, depending on disease stage, pathological risk factors, and patient characteristics [3,4]. Among these modalities, radiotherapy has an indispensable role across multiple clinical settings. External-beam radiotherapy combined with concurrent chemotherapy and intracavitary brachytherapy constitutes the principal curative treatment for locally advanced cervical cancer, while postoperative radiotherapy or chemoradiotherapy is indicated for selected patients with adverse pathological features [5]. Radiotherapy is also used in recurrent and palliative settings [6]. Brachytherapy is particularly important because it enables a high radiation dose to be delivered to the primary tumor while reducing, but not eliminating, exposure to adjacent normal tissues [7]. Owing to the close anatomical relationship between the cervix and vagina, however, vaginal tissues may be included within or located near the treatment volume, making a certain degree of radiation exposure difficult to avoid even with modern image-guided and conformal techniques [8].

Radiation-induced vaginal injury comprises a continuum of acute and chronic tissue abnormalities. Acute manifestations may include mucosal erythema, edema, epithelial desquamation, dryness, discharge, ulceration, bleeding, and pain. When acute epithelial injury persists or tissue repair remains incomplete, progressive vascular dysfunction, chronic inflammation, and stromal remodeling may develop, ultimately leading to mucosal atrophy, loss of elasticity, adhesions, fibrosis, vaginal shortening, and partial or complete stenosis [9,10]. They may cause dyspareunia and sexual dysfunction, negatively affect intimate relationships and psychological well-being, and substantially impair long-term quality of life. Vaginal narrowing and shortening may interfere with pelvic examinations and thereby compromise gynecological surveillance for cancer recurrence; in severe cases, stenosis may also restrict access to the upper vagina and cervix for subsequent clinical procedures [11]. Although the reported incidence varies because of differences in radiation technique, vaginal dose, follow-up duration, toxicity grading, and preventive practices, vaginal injury remains a clinically important and insufficiently addressed consequence of cervical cancer radiotherapy.

Current management relies primarily on vaginal moisturizers and lubricants, topical hyaluronic acid formulations, local hormonal therapy in appropriately selected patients, pelvic-floor rehabilitation, and vaginal dilator therapy [12,13,14,15]. However, these interventions remain largely supportive and are often limited by short residence time, leakage, inconsistent adherence, and the inability to directly modulate the biological cascade of radiation injury.

Polymeric biomaterials offer distinctive opportunities to improve and enhance these methods because their physicochemical, mechanical, and biological properties can be engineered for the moist, acidic, microbially colonized, and mechanically dynamic vaginal environment. Mucoadhesive hydrogels developed for other mucosal tissues provide a potentially transferable strategy for localized vaginal delivery, although their efficacy in irradiated vaginal tissue remains to be established [16]. Polymeric dilators, molds, and stents can be designed with controlled stiffness, reduced surface friction, personalized dimensions, or drug-eluting coatings to improve mechanical support and patient tolerability [17]. For established structural injury, porous scaffolds and tissue-engineered constructs may provide three-dimensional environments that support epithelial repair, vascular remodeling, extracellular-matrix (ECM) organization, and tissue regeneration [18]. Recent advances indicate that the field is beginning to progress from passive hydration and conventional mechanical expansion toward bioactive, personalized, and multifunctional polymer-based interventions.

Existing reviews have predominantly focused on the incidence, pathophysiology, grading, and conventional clinical management of radiation-induced vaginal stenosis [19,20]. Other biomaterials reviews have addressed vaginal drug delivery, gynecological tissue engineering, or polymer-based treatment of cervical tumors, but not the rehabilitation of vaginal tissues following cervical cancer radiotherapy [21,22]. Despite growing interest in post-radiotherapy rehabilitation and polymer-based gynecologic biomaterials, a dedicated synthesis linking the pathological continuum of radiation-induced vaginal injury to stage-specific polymeric interventions is still lacking. Accordingly, this Mini-Review summarizes recent advances in polymeric biomaterials for radiation-induced vaginal injury after cervical cancer radiotherapy, with emphasis on three complementary strategies: local polymeric formulations and mucoadhesive delivery systems, polymer-based dilators and personalized mechanical devices, and regenerative scaffolds and tissue-engineered constructs. By integrating currently fragmented knowledge from radiation oncology, gynecology, polymer science, and regenerative medicine, this review aims to establish a clinically oriented framework for the development of safer, more effective, and patient-centered materials that preserve vaginal structure and function and advance comprehensive women’s health after cervical cancer treatment.

## 2. Pathobiological Basis and Therapeutic Rationale for Polymeric Intervention

**Figure 1:**
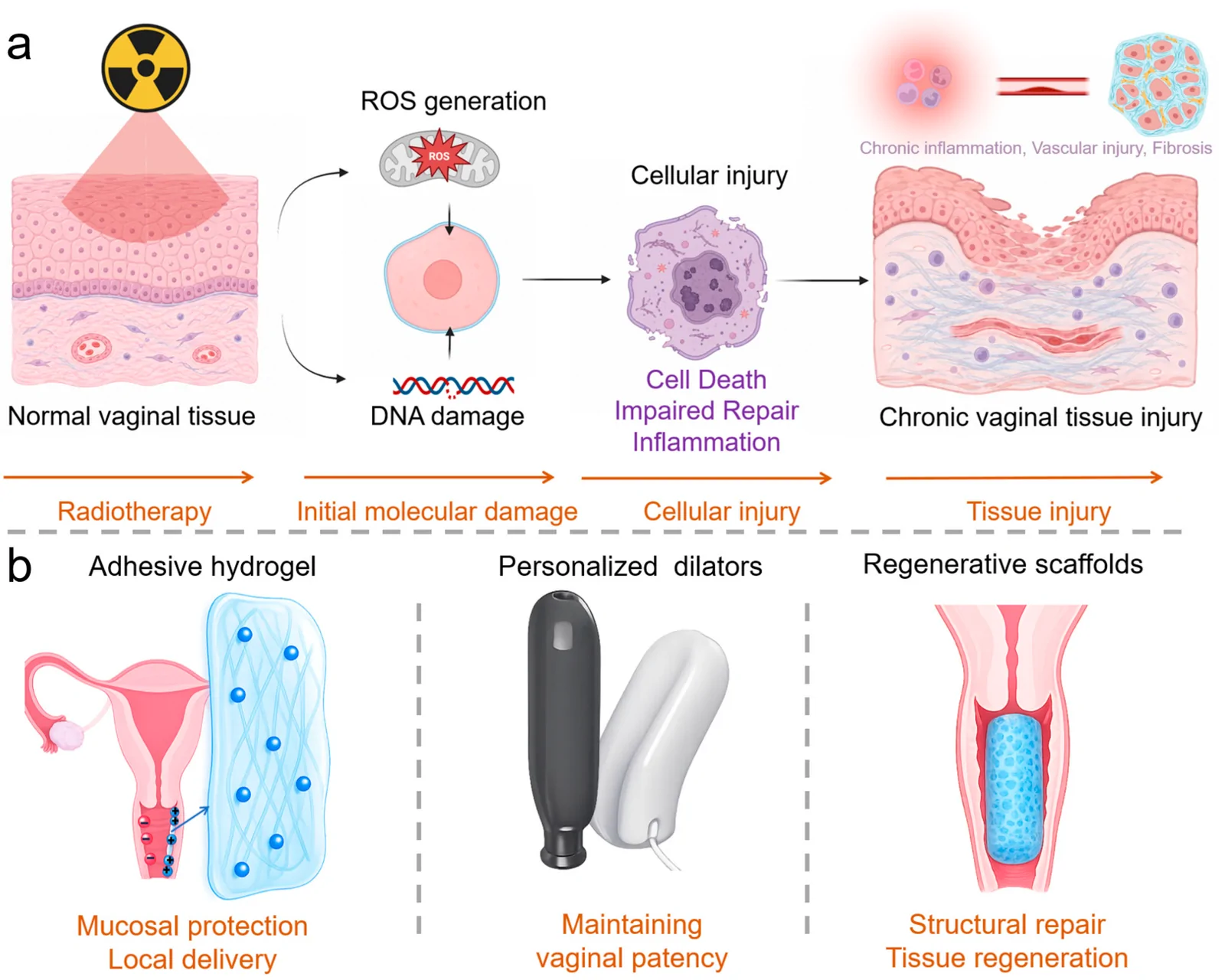
Pathological progression of radiation-induced vaginal injury and corresponding polymeric intervention strategies. (**a**) Radiation-induced molecular and cellular damage progresses toward chronic inflammation, vascular injury, fibrosis, and structural tissue deterioration. (**b**) Polymeric approaches include adhesive hydrogels for mucosal protection and local delivery, personalized dilators for maintaining vaginal patency, and regenerative scaffolds for structural repair and tissue regeneration.

Radiation-induced vaginal injury is a progressive process that extends from initial molecular damage to chronic structural deterioration. As schematized in Figure 1a, ionizing radiation initiates injury through direct energy deposition in DNA and indirect oxidative damage mediated predominantly by reactive oxygen species (ROS) generated through water radiolysis. The resulting oxidative stress disrupts cellular redox homeostasis and damages nuclear DNA as well as mitochondrial DNA, membranes, and respiratory function, thereby further amplifying cellular injury [23,24]. These early molecular events affect multiple compartments of the vaginal wall, including epithelial cells, endothelial cells, fibroblasts, smooth-muscle cells, and resident immune populations. The resulting cellular responses—including apoptosis, proliferative arrest, mitotic failure, and senescence—collectively reduce the regenerative capacity of irradiated tissue.

The vaginal mucosa is particularly vulnerable because maintenance of its stratified squamous epithelium requires continuous renewal from basal and proliferative epithelial-cell populations [25]. Radiation-induced loss or dysfunction of proliferative basal cells impairs epithelial turnover and barrier integrity, leading to epithelial denudation and acute mucositis characterized by erythema, desquamation, friability, ulceration, bleeding, and pain; edema and dryness may develop concurrently as a result of vascular injury and impaired mucosal lubrication [10]. Barrier disruption may further increase susceptibility to friction, microbial perturbation, and repeated mechanical trauma. Although some acute reactions resolve after treatment, persistent oxidative stress, cellular injury, and incomplete re-epithelialization can divert the tissue from physiological healing toward maladaptive repair.

As the injury progresses along the continuum shown in Figure 1a, unresolved inflammation and microvascular dysfunction become increasingly prominent. Pelvic irradiation can reduce vaginal blood flow, whereas radiation-induced endothelial injury and chronic microvascular remodeling may further compromise tissue perfusion, creating a hypoxic and poorly regenerative microenvironment [26]. In parallel, persistent inflammatory signaling and cellular senescence can disrupt normal wound resolution, promote fibroblast activation and myofibroblast differentiation, and drive excessive extracellular-matrix deposition. Together, these interdependent processes shift the irradiated vaginal tissue from incomplete epithelial repair toward progressive fibro atrophic remodeling.

At the tissue level, epithelial atrophy, collagen-rich fibrosis, and disorganization of the elastic-fiber network progressively impair vaginal lubrication, elasticity, wall mobility, and extensibility [27]. Persistent epithelial defects may permit adhesion between opposing mucosal surfaces, whereas continued extracellular-matrix contraction contributes to vaginal shortening, narrowing, and, in severe cases, partial or complete stenosis. Chronic vaginal injury therefore reflects the cumulative interaction of epithelial depletion, impaired vascular support, unresolved inflammation, and pathological matrix remodeling rather than a single isolated mechanism. The severity and anatomical distribution of late vaginal injury are most consistently associated with vaginal radiation dose, irradiated volume, and pre-treatment tumor extension into the vagina. Treatment-related dose distribution and patient-specific factors, including age or estrogen deficiency, hysterectomy status, underlying tissue vulnerability, and adherence to post-treatment vaginal rehabilitation, may further modify the risk [28].

This pathological continuum also provides the rationale for the polymer-based therapeutic framework presented in Figure 1b. Based on the available literature, polymeric interventions can be broadly classified into three complementary categories. First, local polymeric formulations and mucoadhesive hydrogels are designed to protect the injured mucosa, improve hydration, prolong local residence, and enable sustained delivery of antioxidant, anti-inflammatory, hormonal, antifibrotic, or regenerative agents. Second, personalized polymeric dilators, molds, and stents provide controlled mechanical support to separate opposing mucosal surfaces and preserve vaginal patency during tissue remodeling. Third, regenerative polymeric scaffolds and tissue-engineered constructs aim to restore tissue architecture by supporting epithelial regeneration, vascular remodeling, extracellular-matrix organization, and structural reconstruction.

These strategies should not be regarded as rigidly confined to discrete chronological stages. Rather, their therapeutic windows overlap across the evolving injury process, and effective rehabilitation may ultimately require coordinated combinations of mucosal protection, localized biological modulation, mechanical maintenance, and regenerative support. The following sections therefore examine these three polymeric strategies in sequence: local formulations and mucoadhesive delivery systems, personalized mechanical devices, and regenerative scaffolds for tissue reconstruction.

## 3. Polymeric Biomaterial Strategies for Radiation-Induced Vaginal Injury Repair

### 3.1. Local Polymeric Formulations and Mucoadhesive Hydrogels

Current research on local polymeric management of radiation-induced vaginal injury remains limited and heterogeneous. Clinically, most studies have evaluated relatively simple hydrophilic formulations, particularly hyaluronic acid (HA)-containing suppositories or gels, for the relief of dryness, irritation, mucosal inflammation, and pain. By contrast, advanced systems that exploit mucoadhesion, environmental responsiveness, sustained drug release, or tissue-regenerative signaling remain predominantly at the preclinical stage [29]. Moreover, only a small number of biomaterial studies have directly examined irradiated vaginal tissue. Therefore, the evidence discussed below is organized into two complementary tiers: direct studies of radiation-induced vaginal injury and studies in mechanistically related vaginal conditions, such as genitourinary syndrome of menopause and vaginal atrophy. The latter do not provide direct proof of efficacy against radiation injury but reveal material properties and biological functions that may be transferable to the irradiated vaginal microenvironment.

HA is currently the most clinically investigated polymer for radiotherapy-associated vaginal symptoms. Its strong hydration capacity and viscoelasticity allow it to form a temporary lubricating layer over the vaginal epithelium, thereby reducing friction and repeated mechanical irritation. In a randomized study involving 180 women receiving postoperative radiotherapy for cervical cancer, low-molecular-weight HA suppositories reduced acute vaginal symptoms and pain compared with no local treatment [30]. Dinicola et al. similarly reported improved clinical and histological parameters after treatment with a combined formulation containing low-molecular-weight HA and vitamins A and E [31]. In patients undergoing postoperative vaginal brachytherapy for endometrial cancer, Laliscia et al. observed relatively low rates of acute and late vaginal toxicity during treatment with HA-containing ovules, although the absence of an untreated control group precluded definitive conclusions [32]. These studies support the use of HA primarily as a hydrating and surface-protective polymer. However, they do not establish that conventional HA formulations can reverse established collagen accumulation, vaginal shortening, or mature stenosis.

A major limitation of conventional vaginal gels and suppositories is their short and variable residence time caused by mucus turnover, vaginal secretions, gravity, and mechanical activity. Polymeric design can address this problem by integrating rheological control, in situ gelation, and mucosal interactions. For example, Pluronic F127/F68 combined with hydroxypropyl methylcellulose produced shear-responsive vaginal hydrogels that maintained viscosity and mucoadhesion after dilution with simulated vaginal fluid [33]. Amino-functionalization of poloxamer 407 further introduced positively charged groups that interact electrostatically with negatively charged mucins, while preserving thermally induced sol–gel transition [34]. These studies illustrate an important design principle: complementary polymer properties can be combined to balance administration, anatomical conformity, retention, and drug release rather than relying on a single material characteristic.

A recent study provided direct evidence for this strategy in radiation-induced vaginal injury. Zhao et al. developed an amino-terminated Pluronic F127 (AF127) hydrogel carrying fullerenol (FOH) nanoparticles, termed AF127@FOH [35]. Pluronic F127 enabled reversible temperature-dependent gelation, whereas terminal amino groups became protonated in the acidic vaginal environment and enhanced adhesion to negatively charged mucins. The material therefore integrated low-temperature injectability, in situ gel formation, pH-responsive mucoadhesion, and sustained local release. In mice, free fullerenol and unmodified F127@FOH were rapidly cleared, whereas AF127@FOH produced a stronger vaginal fluorescence signal that remained detectable for longer periods. The hydrogel also released fullerenol gradually in simulated vaginal fluid over 24 h. In a 20 Gy acute irradiation model, AF127@FOH preserved epithelial architecture, reduced early collagen deposition and lipid peroxidation, maintained endogenous antioxidant enzyme activities, and partially restored radiation-disrupted vaginal microbial composition [35]. This study is particularly important because its efficacy arose from the coordinated functions of the polymer and its payload: AF127 controlled administration and mucosal retention, whereas fullerenol acted as a stable radical-scavenging effector. Nevertheless, the single-dose acute model does not establish efficacy against chronic vaginal fibrosis or clinically established stenosis.

Because direct regenerative hydrogel studies in irradiated vaginal tissue remain limited, related vaginal atrophy models may provide mechanistic evidence for material design. Although estrogen-deficiency-associated atrophy and radiation-induced vaginal injury are not pathologically equivalent, both involve epithelial thinning, extracellular-matrix remodeling, inflammation, and deterioration of the fibromuscular compartment. Zelus et al. therefore developed a decellularized porcine vaginal extracellular-matrix hydrogel (vECM) as a nonhormonal treatment for genitourinary syndrome of menopause [36].

**Figure 2:**
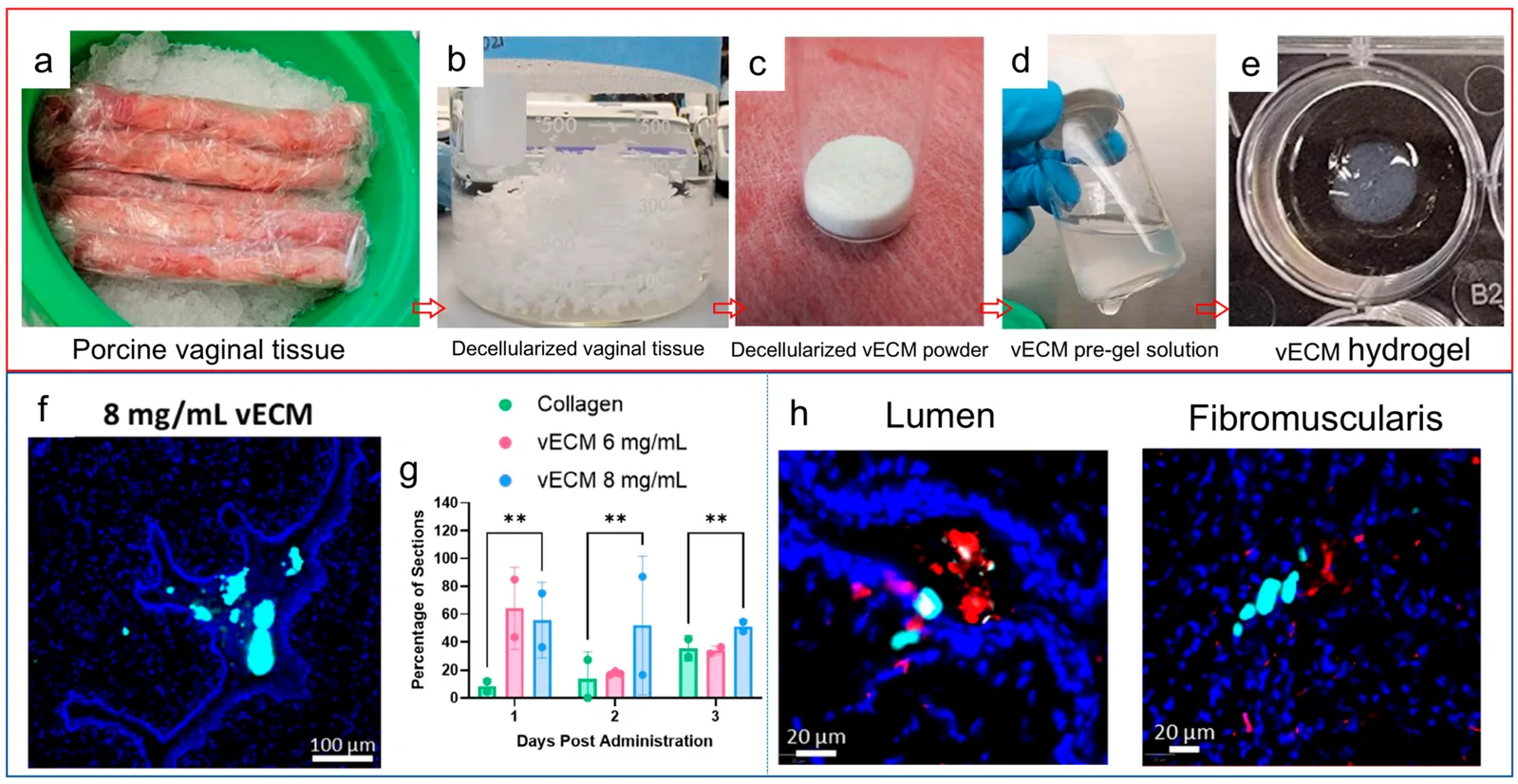
Preparation and intravaginal tissue interactions of porcine vaginal extracellular-matrix hydrogel. (**a**–**e**) Sequential fabrication of vaginal extracellular matrix (vECM) hydrogel, including porcine vaginal tissue harvesting (**a**), decellularization (**b**), lyophilization and milling (**c**), pre-gel preparation (**d**), and hydrogel self-assembly (**e**). (**f**) Representative vaginal tissue section containing fluorescently labeled 8 mg mL^−1^ vECM. (**g**) Percentage of sections containing collagen or vECM within the fibromuscularis at 1–3 days after administration. ** indicates *p* < 0.01. (**h**) Colocalization of vECM with CD68-positive macrophages in the vaginal lumen and fibromuscularis. vECM, cyan; CD68, red; nuclei, blue. Adapted from ref. [36] under the Creative Commons Attribution 4.0 International License.

Figure 2 is included as a material-design example: it illustrates how vaginal tissue-derived extracellular matrix can be processed into an injectable hydrogel and how the formulation distributes within vaginal tissue. The underlying study used a nonirradiated model of vaginal atrophy and therefore provides indirect, rather than direct, evidence for radiation-induced injury. The fabrication sequence is summarized in Figure 2a–e. Native porcine vaginal tissue was gently decellularized to remove cellular components while retaining tissue-specific ECM constituents (Figure 2a,b). The decellularized tissue was subsequently lyophilized and milled into vECM powder (Figure 2c), followed by enzymatic digestion and neutralization to generate a syringe-deliverable pre-gel solution (Figure 2d) capable of self-assembling into a hydrogel under physiological conditions (Figure 2e). Compared with the 6 mg mL^−1^ formulation, the 8 mg mL^−1^ vECM exhibited greater viscosity at low shear rates and more rapid self-assembly while retaining shear-thinning behavior compatible with intravaginal administration [36].

The short-term tissue distribution of the formulation further indicates that vECM does not function exclusively as a luminal coating. Following intravaginal administration, fluorescently labeled 8 mg mL^−1^ vECM was detected within vaginal tissue sections (Figure 2f) and persisted within the fibromuscularis for up to 3 days (Figure 2g). The overlap between the vECM-associated fluorescence and cluster of differentiation (CD) 68-positive cells suggests spatial proximity to host macrophages (Figure 2h). However, colocalization alone does not establish cellular uptake, macrophage polarization, or a causal immunomodulatory effect [36].

After 14 days of treatment in ovariectomized rats, 8 mg mL^−1^ vECM increased epithelial thickness and differentiation, smooth-muscle thickness and proliferation, and the proportion of CD163-positive M2-like macrophages [36]. These effects correspond to several therapeutic requirements of radiation-induced vaginal injury, particularly restoration of epithelial integrity, preservation of the fibromuscular compartment, and regulation of chronic inflammation. However, the model did not reproduce radiation-specific vascular injury, oxidative stress, or progressive fibrosis. Thus, this study should be regarded as a design precedent for tissue-instructive vaginal hydrogels rather than direct evidence of efficacy after radiotherapy. Future studies using fractionated irradiation models should determine whether similar systems can preserve tissue compliance, regulate collagen remodeling, and ultimately prevent vaginal adhesion and stenosis. The potential advantage of vECM over platelet-rich plasma (PRP)-based gels lies primarily in tissue specificity rather than in a greater abundance of soluble growth factors. vECM provides a complex matrix of collagens, glycoproteins, proteoglycans, and matrix-associated signals derived from vaginal tissue, thereby supplying adhesion sites and biochemical cues potentially relevant to epithelial, stromal, smooth-muscle, and immune-cell responses. By comparison, PRP provides an autologous fibrin network containing concentrated growth factors and is more readily obtained, but its composition depends on the donor and preparation protocol, its growth-factor release is activation dependent and relatively transient, and it does not reproduce the tissue-specific matrix environment of the vagina. Conversely, vECM is xenogeneic, its composition may be altered by decellularization and enzymatic digestion, and its manufacturing reproducibility and long-term safety require further evaluation. PRP has shown preliminary benefits in vulvovaginal atrophy, but neither platform has demonstrated efficacy against chronic radiation-induced vaginal fibrosis or stenosis [37]. Therefore, the available evidence does not establish the superiority of vECM over PRP. Direct comparisons should assess retention, epithelial and vascular repair, immune responses, collagen organization, tissue compliance, and prevention of stenosis.

Taken together, the available evidence illustrates a progression from passive hydration toward active and tissue-instructive local therapies. Conventional HA formulations primarily provide hydration and surface protection; AF127@FOH demonstrates how mucoadhesion and responsive gelation can enable sustained local radioprotection; and vECM suggests that tissue-specific matrices may additionally regulate immune responses and repair across multiple vaginal layers. Future formulations should integrate atraumatic mucosal retention with antioxidant, regenerative, or antifibrotic activity and should be evaluated in fractionated irradiation models using endpoints that include epithelial recovery, vascular integrity, collagen organization, smooth muscle preservation, tissue compliance, adhesion formation, and stenosis.

### 3.2. Polymeric Personalized Vaginal Dilators

Mechanical rehabilitation represents a particularly relevant application of polymeric biomaterials for radiation-induced vaginal injury. Unlike local hydrogels, which primarily regulate the mucosal microenvironment, vaginal dilators and stents provide radial mechanical support to oppose circumferential contraction and preserve vaginal patency [17]. Polymers are especially suitable for this purpose because their mechanical behavior, surface properties, and processing characteristics can be tailored through molecular structure, crosslinking, composition, and device design [38]. Rigid thermoplastics can provide dimensional stability and reproducible size progression, whereas elastomers and shape-memory polymers can enable compliant tissue contact, large reversible deformation, compact insertion, and gradual expansion after placement [39,40]. Taken together, these tunable material properties make polymers particularly well suited to the fabrication of vaginal dilators, because device stiffness, insertion profile, expansion behavior, and tissue conformity can be tailored to balance effective radial support with atraumatic contact.

Rigid thermoplastics remain the most clinically accessible option. Kongwattanakul et al. evaluated 3D-printed Polylactic acid (PLA) dilators with diameters of 2.0, 2.5, and 3.0 cm in 67 women treated for cervical cancer [41]. Most patients developed grade 1 stenosis, whereas 15.79% developed grade 2 stenosis; however, only 37 completed follow-up, vaginal narrowing persisted, and some users considered the devices overly stiff. Because the dilators were covered with condoms during use, the study mainly supports the feasibility, affordability, and standardized sizing of 3D-printed devices rather than any specific therapeutic benefit of PLA itself.

**Figure 3:**
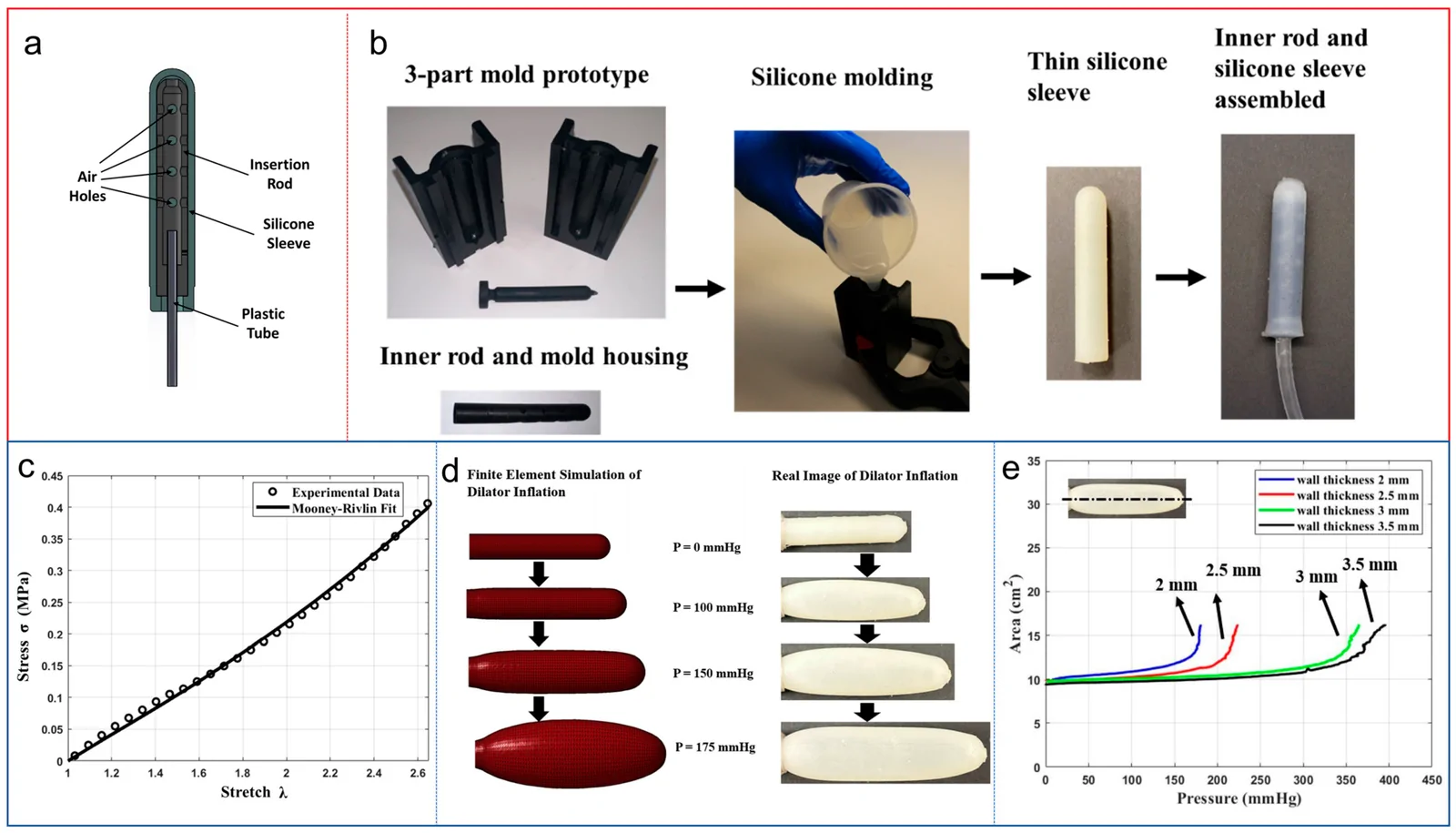
Design, fabrication, and mechanical characterization of an inflatable silicone vaginal dilator. (**a**) Device architecture comprising a rigid insertion rod, air-supply tube, air outlets, and an outer silicone sleeve. (**b**) Fabrication process using a three-part 3D-printed mold, silicone casting, sleeve demolding, and device assembly. (**c**) Experimental stress–stretch response of the silicone elastomer and corresponding Mooney–Rivlin model fit. (**d**) Finite-element simulations and experimental images showing pressure-dependent expansion of a dilator with a 2 mm wall thickness. (**e**) Experimental relationships between internal pressure and longitudinal cross-sectional area for silicone sleeves with wall thicknesses of 2.0–3.5 mm. Adapted from ref. [42] under the Creative Commons Attribution 4.0 International License.

Elastomeric polymers offer a route to replace fixed-diameter rigid dilators with compliant, pressure-controlled devices. Figure 3 is included to show how polymer selection and sleeve geometry can be used to control the expansion of an inflatable vaginal dilator, rather than to demonstrate clinical efficacy. Chen et al. developed an inflatable vaginal dilator for radiotherapy-induced vaginal stenosis, comprising a rigid insertion rod, an air-supply tube, and an outer sleeve molded from Dragon Skin 10 Medium silicone elastomer (Figure 3a) [42]. The silicone sleeve was fabricated using a three-part 3D-printed mold and subsequently assembled around the internal rod (Figure 3b). Mechanical testing demonstrated a nonlinear hyperelastic stress–stretch response that was well described by the Mooney–Rivlin model (Figure 3c). Using the experimentally derived material parameters, finite-element simulations reproduced the progressive expansion of a 2 mm-thick sleeve with increasing internal pressure and agreed closely with the observed device deformation (Figure 3d). Experimental pressure–expansion curves further showed that thicker silicone walls required higher pressures to achieve a comparable cross-sectional area (Figure 3e) [42]. These findings demonstrate that both the constitutive behavior of the elastomer and the structural geometry of the sleeve can be used to tune dilator expansion. This design permits insertion at a relatively small initial diameter followed by gradual radial enlargement, potentially improving conformity and avoiding abrupt transitions between rigid dilator sizes. These measurements establish material behavior and device-level expansion under experimental conditions. The internal inflation pressures shown in Figure 3e cannot be assumed to equal the contact pressures experienced by the vaginal wall. Contact-pressure distribution, repeated-use durability, frictional injury to irradiated mucosa, patient tolerability, and clinical effectiveness remain to be evaluated. The clinical evidence should also be distinguished from the engineering feasibility of these devices. A Cochrane review found no reliable evidence that routine vaginal dilation during radiotherapy prevents stenosis, although observational studies suggest a possible association between post-radiotherapy dilation and a lower incidence of stenosis [43]. Furthermore, a systematic review identified 195 potentially relevant records but found no eligible studies directly comparing plastic and silicone dilators in terms of efficacy, cost, or patient experience [44]. An automated expandable dilator prototype has also been developed to regulate pressure and gradually increase vaginal expansion; however, its validation was limited to modeling and benchtop experiments [45]. These findings indicate that advanced polymer design may improve conformability and pressure control, but comparative clinical evidence demonstrating superiority over conventional dilators remains unavailable.

Related cervical-device research further illustrates the importance of polymer-controlled stress distribution. Zou et al. designed a fluid–solid coupled vaginal dilation system using hyperelastic medical-grade silicone rubber for cervical examination [46]. Compared with a conventional rigid duckbill device, the flexible–rigid system increased the tissue-contact area and produced a more uniform pressure distribution in a simulated vaginal model. Although this device was designed for colposcopy rather than post-radiotherapy rehabilitation, it supports a transferable engineering principle: compliant polymer interfaces may reduce local stress concentrations while maintaining sufficient overall expansion. Whether such pressure redistribution can improve tolerance and adherence in women with fragile irradiated mucosa remains to be tested.

A more advanced approach is to encode deployment and anatomical adaptation directly into the polymer network. Hicks et al. developed a self-fitting vaginal stent from polycaprolactone (PCL)-based shape-memory polymer foam for the prevention of stenosis after pelvic radiotherapy or vaginal reconstruction [17]. Two photocurable macromolecular architectures—linear PCL diacrylate and star-shaped PCL tetraacrylate—were compared. The star-shaped architecture reduced the melting transition into a range compatible with physiological activation and enabled approximately 84% shape recovery at 37 °C, whereas the linear network showed inadequate recovery under the same conditions. Emulsion templating generated highly porous polymerized high-internal-phase-emulsion foams with shape fixity exceeding 90% [17]. The star-PCL foam was subsequently fabricated into a hollow cylindrical stent that could be radially compressed by approximately 50% to an outer diameter of around 11 mm for insertion. The complete device exhibited greater than 95% shape fixity in its compressed state and nearly complete radial recovery after activation. In a benchtop pelvic model, irrigation with warm water triggered expansion against the simulated vaginal walls within 5 min, increasing the cross-sectional area by approximately 70% and maintaining vaginal caliber under simulated physiological pressures. Its hollow geometry permitted the egress of vaginal secretions, while the porous and potentially degradable PCL network offers a fundamentally different design from nonporous rigid rods. The material remained relatively stable during the reported six-week degradation assessment, with less than 2% mass loss under neutral and acidic conditions, indicating suitability for temporary rather than rapidly resorbing support.

Evidence from vaginal reconstruction provides an additional precedent for combining polymers with complementary mechanical functions. Coskun et al. reported a composite vaginal stent comprising a rigid acrylic core covered by a silicone layer [47]. The acrylic component-maintained geometry and resisted contraction, whereas the softer silicone surface provided a more compliant tissue-contacting interface. Although this evidence arose from vaginoplasty rather than radiation injury, the rigid-core/soft-interface concept remains relevant to the design of dilators for irradiated vaginal tissue, where adequate mechanical support must be balanced against mucosal fragility.

Overall, polymeric vaginal devices are evolving from fixed, rigid cylinders toward compliant, self-expanding, and anatomically adaptive systems. Future development should treat polymer modulus, shape-recovery temperature, radial force, wall thickness, porosity, surface friction, fatigue resistance, and fluid drainage as quantifiable design parameters rather than secondary manufacturing details. Multimaterial structures may combine a load-bearing core with a low-friction compliant surface, while porous or coated devices could potentially integrate mechanical dilation with local delivery of lubricating, anti-inflammatory, or antifibrotic agents. This latter concept remains hypothetical and should not be presented as clinically established. Direct comparisons in fractionated irradiation models and prospective clinical studies will be required to determine whether advanced polymer architectures improve vaginal compliance, mucosal safety, patient adherence, and long-term stenosis outcomes relative to conventional rigid dilators.

### 3.3. Regenerative Scaffolds and Tissue-Engineered Constructs

Unlike vaginal dilators and mechanical support devices, which primarily preserve luminal patency, regenerative scaffolds are designed to participate directly in tissue reconstruction. By providing a temporary three-dimensional framework, these constructs may facilitate epithelial coverage, vascular ingrowth, ECM remodeling, and smooth-muscle regeneration, while also serving as local carriers for cells, extracellular vesicles, or therapeutic factors. Because direct evidence on scaffold-mediated repair of radiotherapy-induced vaginal injury remains limited, we also considered studies of congenital vaginal agenesis, pelvic-organ-prolapse models, and surgically created vaginal defects. Although these conditions differ from radiation injury, they involve overlapping regenerative requirements, including epithelial restoration, vascular ingrowth, extracellular-matrix remodeling, smooth-muscle regeneration, and maintenance of luminal patency, and therefore provide relevant indirect evidence for scaffold design.

Ye et al. implanted porous protein scaffolds loaded with adipose-derived mesenchymal stem cells into irradiated rat vaginal tissue and reported enhanced epithelial regeneration, increased expression of proliferative and mucosal markers, and improvement in stenosis and contracture compared with less complex treatments [48]. This study provided initial proof that three-dimensional scaffolds can enhance the retention and regenerative effects of transplanted cells in an irradiated vaginal environment. Nevertheless, the treatment groups were extremely small, follow-up was short, and long-term endpoints—including vascular perfusion, collagen and elastin organization, circumferential compliance, scaffold degradation, luminal patency, and recurrent stenosis—were not adequately assessed. The study therefore supports feasibility but does not establish durable reversal of radiation-induced vaginal fibrosis.

The general feasibility of scaffold-mediated vaginal reconstruction was initially demonstrated using synthetic biodegradable polymers. De Filippo et al. seeded rabbit vaginal epithelial and smooth-muscle cells onto polyglycolic acid scaffolds, producing vascularized constructs with organized epithelial and muscular layers and contractile responses resembling those of native vaginal tissue [49]. The same group subsequently used autologous vaginal cells and biodegradable tubular scaffolds to achieve complete vaginal replacement in rabbits; after six months, the reconstructed vaginas remained patent and showed organized tissue layers and physiological contractility [50]. This approach was later translated into a pilot clinical study involving four patients with Mayer–Rokitansky–Küster–Hauser syndrome, in whom autologous cell-seeded biodegradable scaffolds maintained structural and functional variables during follow-up of up to eight years [51]. These studies established the biological feasibility of polymer-supported vaginal tissue formation, although their relevance to radiotherapy survivors is limited by the relatively healthy recipient beds and the complexity of individualized cell manufacturing. The rationale for epithelial-cell seeding requires qualification. The vaginal constructs described above did not rely on terminally differentiated superficial epithelial cells; instead, vaginal epithelial cells were isolated from small tissue biopsies, expanded ex vivo, and subsequently seeded onto scaffolds [49,50,51,52]. Their intended function is to accelerate formation of a continuous luminal barrier, thereby reducing exposure of the underlying scaffold, frictional injury, adhesion formation, and infection. Nevertheless, their clonogenic capacity and long-term engraftment have not been established in irradiated vaginal tissue. Future studies should therefore characterize the proliferative or progenitor-cell fraction before implantation and determine whether the seeded cells persist or are eventually replaced by host-derived epithelium.

Synthetic elastomeric scaffolds provide greater control over pore architecture, degradation, and mechanical behavior. Sartoneva et al. developed a supercritical-carbon-dioxide-foamed poly-L-lactide-co-ε-caprolactone scaffold with approximately 65% porosity, a mean pore size of approximately 350 μm, and an elastic modulus of approximately 2.8 MPa [52]. Human vaginal epithelial and stromal cells adhered to the scaffold, remained viable, and retained relevant phenotypic markers during short-term culture. Nevertheless, this evidence remains limited to in vitro conditions, and the wet-state friction, cyclic deformation, degradation, and mechanical compatibility of these scaffolds with irradiated vaginal tissue remain unknown.

**Figure 4:**
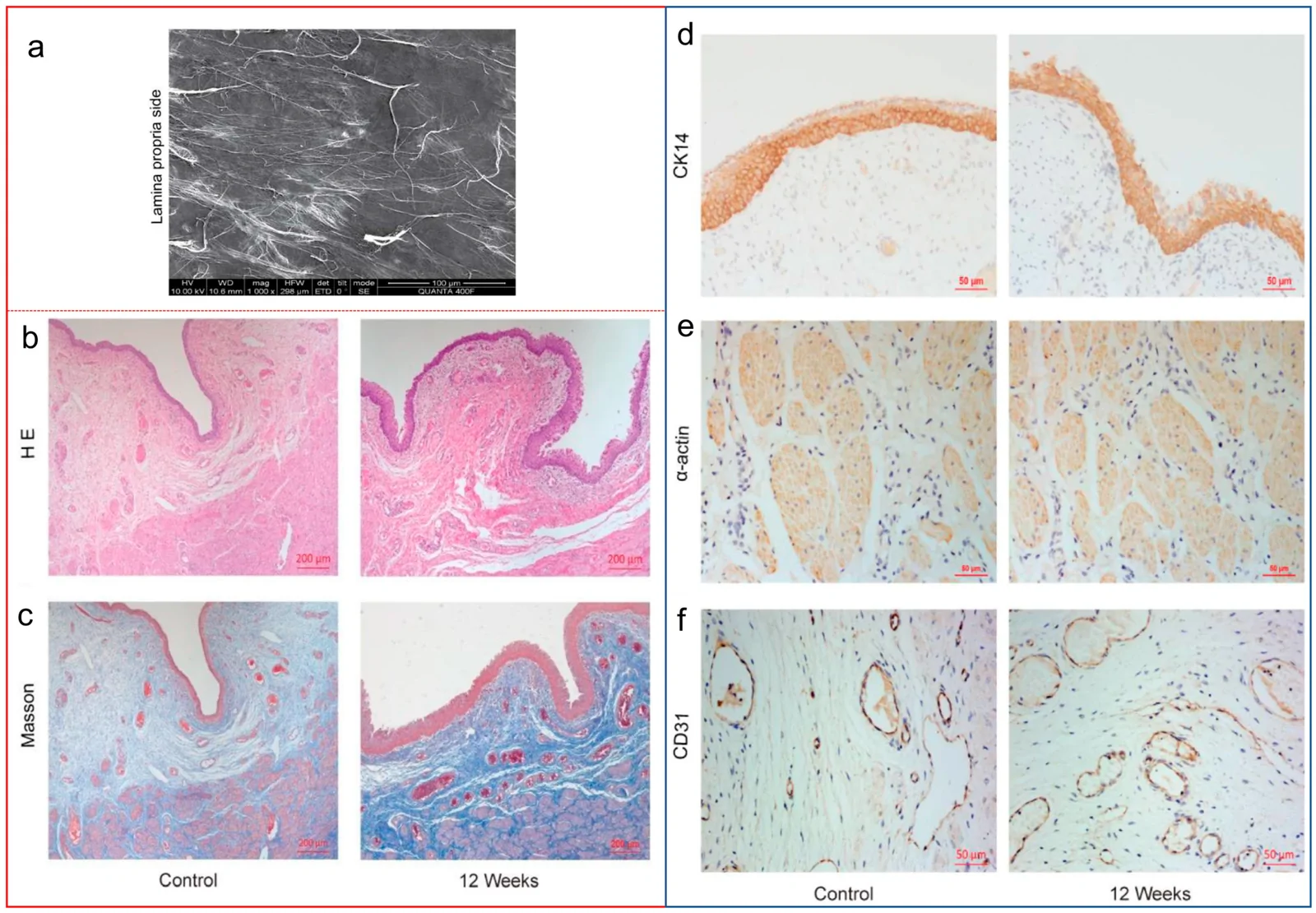
SIS-supported vaginal reconstruction in a non-irradiated minipig segmental-defect model. (**a**) Scanning electron micrograph of the lamina propria surface of SIS. Representative hematoxylin and eosin (H&E) (**b**), Masson’s trichrome (**c**), CK14 (**d**), α-actin (**e**), and CD31 (**f**) staining of normal vaginal tissue and reconstructed neovagina 12 weeks after implantation, illustrating restoration of epithelial, stromal, smooth-muscle, and vascular components. Scale bars: 100 μm in (**a**), 200 μm in (**b**,**c**), and 50 μm in (**d**–**f**). Adapted from Xiao et al. [53] under the Creative Commons Attribution 4.0 International License.

Natural biopolymeric and ECM-derived scaffolds provide a complementary regenerative strategy by preserving collagen-rich architectures and matrix-associated biological cues. Xiao et al. evaluated a multilayered small-intestinal-submucosa (SIS) scaffold in a minipig model in which an approximately 4-cm full-thickness vaginal segment was resected and replaced with the graft [53]. SIS is not a synthetic polymer; rather, it represents an ECM-derived natural biopolymeric scaffold whose structural framework is dominated by collagen. Scanning electron microscopy of the lamina propria surface revealed an interwoven fibrous network that could provide a permissive substrate for host–cell attachment and infiltration (Figure 4a). At 12 weeks after implantation, hematoxylin and eosin staining showed restoration of a multilayered mucosal epithelium and underlying stromal organization compared with normal vaginal controls (Figure 4b), whereas Masson’s trichrome staining demonstrated more organized smooth-muscle bundles and ECM architecture within the reconstructed tissue (Figure 4c). Immunohistochemical comparisons between normal controls and the 12-week neovagina further showed cytokeratin 14 (CK14)-positive epithelial coverage, α-actin-positive smooth-muscle structures, and CD31-positive vascular profiles, supporting concurrent epithelial, muscular, and vascular regeneration (Figure 4d–f). Collectively, these findings demonstrate that a collagen-rich ECM scaffold can support multicompartment vaginal tissue reconstruction in a clinically relevant large-animal segmental-defect model. However, because the study used a non-irradiated surgical-defect model and did not include an untreated defect group, the specific contribution of SIS cannot be fully separated from spontaneous repair, and its efficacy in the hypovascular and fibrotic microenvironment of radiation injury remains unproven. Likewise, the reported association with Hippo/yes-associated protein–transcriptional coactivator with PDZ-binding motif (YAP–TAZ) signaling should be regarded as hypothesis-generating rather than causal, as no pathway inhibition or genetic validation was performed [53].

Organ-specific ECM may provide more appropriate biochemical and structural cues than SIS. Tian et al. prepared an acellular vaginal matrix from porcine tissue and implanted it into vaginal defects in Bama miniature pigs [54]. The regenerated tissues developed mucosal folds, epithelial and smooth-muscle components, and partial recovery of electrophysiological properties. However, estrogen-receptor expression remained lower than that in native vaginal tissue, indicating that morphological reconstruction did not fully restore tissue-specific endocrine function [54]. Cell loading may further enhance the biological activity of ECM scaffolds. In ovariectomized rhesus monkeys, Ma et al. seeded human umbilical-cord mesenchymal stromal cells onto SIS grafts and observed enhanced ECM reorganization, angiogenesis, muscle-bundle formation, and vaginal mechanical properties after implantation [55]. Nevertheless, pelvic-organ-prolapse and estrogen-deficiency models do not reproduce the endothelial injury, chronic oxidative stress, and progressive fibrosis associated with radiotherapy.

Three-dimensional bioprinting enables tissue-specific ECM to be combined with mechanically tunable polymers and spatially organized biological cargo. Hou et al. formulated porcine acellular vaginal matrix with gelatin and sodium alginate to produce a printable bioink containing bone-marrow-derived mesenchymal stromal cells [56]. The constructs supported cell survival and promoted vascularization and epithelial-like differentiation after subcutaneous implantation, although the ectopic model did not reproduce the luminal and mechanical environment of the vagina. Shi et al. subsequently combined vaginal decellularized extracellular matrix (dECM), gelatin methacryloyl, and silk fibroin in printed tubular scaffolds containing mesenchymal-stromal-cell-derived exosomes [57]. These constructs provided sustained exosome release and promoted epithelial, vascular, and smooth-muscle regeneration in a rat vaginal-reconstruction model. More recently, Zhang et al. evaluated bone marrow-derived mesenchymal stromal cells (BMSC)-loaded vECM–gelatin methacryloyl (GelMA)–silk-fibroin scaffolds in rabbits and used integrated transcriptomic and proteomic analyses to implicate ECM remodeling, inflammatory regulation, angiogenesis, epithelialization, and muscle formation [58]. These studies illustrate the design flexibility of hybrid polymer–ECM constructs, but the proposed molecular mechanisms remain largely associative and have not been validated in irradiated vaginal tissue. The rationale for mesenchymal stromal cell loading is based primarily on their paracrine, immunomodulatory, proangiogenic, and trophic effects rather than an assumption of durable differentiation into vaginal tissue. Nevertheless, their phenotype is microenvironment dependent. In a transforming growth factor-β (TGF-β)-rich and mechanically abnormal fibrotic environment, mesenchymal stromal cells may acquire fibroblastic or myofibroblastic characteristics and potentially aggravate matrix deposition. Cell-loaded scaffolds should therefore be evaluated by cell-fate tracking and longitudinal assessment of α-smooth-muscle actin, collagen organization, tissue contraction, and vaginal compliance. Direct comparisons among acellular scaffolds, stromal-cell-loaded scaffolds, and cell-free products such as extracellular vesicles are also required to determine whether cellular implantation provides benefits that justify its fibrotic and oncological risks [59].

Vascularization is likely to be a decisive barrier in irradiated tissues. Jakubowska et al. generated a prevascularized, scaffold-free human vaginal mucosal construct containing vaginal fibroblasts, endothelial cells, and epithelial cells [60]. Following implantation in mice, the engineered endothelial networks connected with host vessels and contained host erythrocytes, demonstrating functional inosculation. Although this construct was not developed for radiation injury, it indicates that preformed microvascular networks may improve graft perfusion before extensive host-vessel ingrowth occurs. Such strategies may be especially important in irradiated tissue, where baseline perfusion is already compromised.

Several design criteria are particularly important for scaffolds intended to repair radiation-induced vaginal injury. An increase in collagen staining should not, by itself, be interpreted as successful regeneration, because irradiated vaginal tissue is characterized by dense collagen deposition, abnormal elastin organization, fibrosis, and loss of compliance [19,27]. Regenerative outcomes should therefore be evaluated using a combination of epithelial integrity, vascularization, collagen density and organization, elastin morphology, matrix turnover, wet-state mechanical compliance, and sustained luminal caliber, rather than total collagen content alone. By analogy with other tubular and bilayer tissue-engineering systems, an optimal vaginal scaffold may benefit from a relatively dense and smooth luminal layer that supports continuous epithelialization, together with a more porous outer compartment that permits host–cell infiltration, nutrient transport, and vascular ingrowth [61,62]. This architecture remains a design hypothesis for irradiated vaginal tissue and requires direct experimental validation.

Scaffold degradation must also be coordinated with the rate of tissue maturation. Premature degradation may remove mechanical support before the regenerated tissue becomes structurally competent, whereas excessively slow degradation may restrict tissue replacement and prolong chronic inflammation, foreign-body reactions, and fibrous encapsulation [63,64]. In addition, constructs containing living mesenchymal stromal cells or strongly mitogenic, immunomodulatory, or proangiogenic signals require careful oncological assessment before use in cervical-cancer survivors, because several processes that support tissue repair—including revascularization, cell survival, and immunomodulation—may also interact with residual or dormant malignant cells [65]. Overall, regenerative scaffolds remain a promising but predominantly preclinical strategy for radiotherapy-induced vaginal injury. A clinically realistic near-term approach may be an acellular or cell-free hybrid scaffold combining tissue-specific ECM cues with a degradable synthetic polymer network and controlled delivery of pro-epithelial, vascular-supportive, or antifibrotic agents. Future studies should move beyond clean surgical-defect models and evaluate these systems in chronic irradiation models that reproduce epithelial atrophy, vascular rarefaction, collagen and elastin disorganization, matrix contraction, and reduced tissue compliance. At present, vaginal tissue-engineering studies demonstrate biological feasibility, but they do not yet establish durable treatment of radiation-induced vaginal stenosis.

## 4. Future Perspectives

Polymeric strategies for radiation-induced vaginal injury have progressed from passive formulations toward responsive delivery systems, adaptive devices, and regenerative constructs. However, direct evidence remains sparse and is distributed across acute irradiation models, non-irradiated reconstruction studies, and engineering prototypes. Future work should therefore prioritize pathobiology-aligned intervention, mechanism-based material innovation, and clinically relevant validation.

### 4.1. Stage-Specific and Multifunctional Interventions

Interventions should be matched to the dominant pathological stage rather than applied uniformly throughout the injury course. Early treatment should emphasize hydration, epithelial-barrier preservation, and control of oxidative and inflammatory injury, whereas established fibroatrophic remodeling is more likely to require antifibrotic modulation combined with mechanical maintenance or structural reconstruction. Because these therapeutic windows overlap, modular systems may be more appropriate than single-function products. Examples include compliant dilators with lubricating or drug-eluting interfaces and bilayer scaffolds combining a smooth epithelializing surface with a porous vascular-supportive compartment [42,61,62]. Such integration should be driven by functional complementarity rather than maximal complexity. Drug release, wet-state friction, radial force, degradation, drainage, and tissue compatibility must be co-optimized to avoid exchanging biological benefit for mechanical trauma or treatment burden.

A practical research priority is to compare a small number of platforms against current care, each addressing a defined limitation: a mucoadhesive antioxidant hydrogel versus a conventional hyaluronic acid formulation for local retention and early mucosal protection; a compliant, pressure-controlled dilator versus a rigid dilator for tissue contact and patient tolerability; and an acellular vaginal extracellular-matrix–polymer scaffold for persistent structural defects. These approaches should be evaluated according to their intended functions rather than a common measure of epithelial thickness alone. Relevant outcomes include local residence and epithelial recovery for hydrogels, contact pressure and mucosal injury for dilators, and perfusion, matrix organization, compliance, and sustained luminal patency for scaffolds. Future development should therefore focus on modular bio-implants in which the polymer platform and biological cargo are matched to the stage of injury. During early mucosal injury, mucoadhesive hydrogels could combine prolonged vaginal retention with controlled delivery of antioxidant, anti-inflammatory, antifibrotic, or pro-epithelial agents. During tissue remodeling, compliant dilators or stents with lubricating and drug-eluting interfaces could couple maintenance of vaginal patency with local biological modulation. For established structural damage, acellular vaginal-ECM–polymer scaffolds may provide both mechanical support and tissue-specific regenerative cues. Cellular products, particularly extracellular vesicles or defined secretomes, may retain some paracrine benefits of living cells while reducing concerns related to cell persistence, uncontrolled differentiation, fibrosis, and oncological safety. Living-cell-loaded constructs should be advanced only when they demonstrate a reproducible advantage over acellular or cell-free alternatives. For all of these systems, future studies must define release kinetics, biological potency, degradation, sterilization, manufacturing reproducibility, and long-term functional outcomes.

### 4.2. Emerging Materials and Bioactive Payloads

Carbon dots and curcumin are considered here as exploratory payloads for the delivery platforms discussed above, rather than stand-alone solutions for vaginal fibrosis or stenosis. Emerging components should be selected according to defined radiation-associated mechanisms rather than material novelty alone. Carbon dots are a representative candidate because selected formulations possess ROS-scavenging, anti-inflammatory, and fluorescence-tracking properties. A carbon-dot nanoenzyme hydrogel has recently demonstrated antioxidant and anti-inflammatory activity at a mucosal surface [66]. However, carbon dots are not a uniform material class; their surface chemistry and synthesis conditions can determine whether they scavenge or generate ROS [67]. Their use in irradiated vaginal tissue therefore requires formulation-specific evaluation of retention, clearance, microbiome interactions, and repeated-dose mucosal safety.

Curcumin is better regarded as a repurposed bioactive payload than as a new drug. Intravaginal curcumin nanoparticles have reduced local inflammatory responses in experimental female reproductive-tract inflammation, while topical or nanoformulated curcumin has shown preliminary benefit in radiation-induced oral mucositis [68,69]. Its poor aqueous solubility and limited stability favor a two-level delivery strategy in which curcumin is first incorporated into nanoparticles, micelles, liposomes, or inclusion complexes and then retained locally within a mucoadhesive hydrogel. Neither carbon dots nor curcumin has yet demonstrated efficacy against chronic radiation-induced vaginal fibrosis or stenosis. Their development should therefore include direct testing in irradiated vaginal tissue and assessment of possible effects on residual cervical-cancer cells and tumor radiosensitivity.

### 4.3. Preclinical Validation and Clinical Translation

The principal translational limitation is the mismatch between available models and the chronic clinical phenotype. Single-dose acute irradiation and non-irradiated surgical-defect models provide useful mechanistic information but do not fully reproduce fractionated radiation exposure, vascular rarefaction, persistent inflammation, matrix contraction, and progressive loss of compliance [26,35,60]. Future studies should incorporate localized fractionated irradiation, extended follow-up, and clinically relevant modifiers such as estrogen deficiency and mechanical rehabilitation.

Evaluation should extend beyond epithelial thickness and total collagen staining. Constructive repair should be defined through epithelial integrity, perfusion, collagen organization and subtype balance, elastin architecture, matrix turnover, smooth-muscle preservation, wet-state compliance, adhesion formation, and sustained luminal caliber. Clinical studies should additionally include pain, bleeding, dilator tolerance, sexual function, adherence, quality of life, and feasibility of gynecological surveillance.

Oncological safety is particularly important for cell-, extracellular-vesicle-, growth-factor-, or proangiogenic systems because regenerative signals may also interact with residual or dormant malignant cells [65,70]. In the near term, acellular hydrogels, cell-free ECM–polymer composites, and removable drug-eluting devices may offer the most realistic balance among biological activity, manufacturability, controllability, and safety. Translation will still require reproducible manufacturing, sterilization, storage stability, degradation-product assessment, and repeated-dose local toxicity testing. This concern does not establish that mesenchymal-stromal-cell-loaded vaginal scaffolds promote cervical cancer recurrence. It does, however, justify testing whether their secreted factors affect residual cancer-cell survival or growth before clinical use, alongside assessment of their regenerative benefits. Acellular and cell-free platforms may therefore provide a more readily testable starting point for translation.

## 5. Conclusions

The studies reviewed here suggest three complementary roles for polymeric biomaterials in radiation-induced vaginal injury: local mucosal protection and delivery, mechanical maintenance of vaginal patency, and structural tissue repair. Hyaluronic acid formulations have been evaluated in patients receiving pelvic radiotherapy and may relieve vaginal symptoms, but their ability to prevent or reverse established stenosis remains unproven. The AF127@FOH hydrogel improved local retention and reduced early tissue injury in an acute irradiation model, whereas vaginal extracellular-matrix hydrogels promoted epithelial and fibromuscular repair in nonirradiated atrophy models. The latter findings provide a rationale for material design but cannot yet be taken as evidence of efficacy after radiotherapy.

Polymeric dilators and stents demonstrate increasingly controllable expansion and anatomical adaptation, although clinical superiority over conventional devices has not been established. Regenerative scaffolds have supported vaginal tissue formation in reconstruction models, with limited preliminary evidence in irradiated tissue; durable restoration of vascularization, tissue compliance, and luminal patency remains uncertain. The next step is to compare clinically practical hydrogels, compliant devices, and acellular scaffolds with existing care in fractionated irradiation models, using functional outcomes and oncological safety alongside histological measures. This distinction between demonstrated benefit and plausible application should guide translation to cervical cancer survivors.

## Data Availability

The original contributions presented in the study are included in the article; further inquiries can be directed to the corresponding author.
